# Beetroot, A Remarkable Vegetable: Its Nitrate and Phytochemical Contents Can be Adjusted in Novel Formulations to Benefit Health and Support Cardiovascular Disease Therapies

**DOI:** 10.3390/antiox9100960

**Published:** 2020-10-08

**Authors:** Diego dos S. Baião, Davi V. T. da Silva, Vania M. F. Paschoalin

**Affiliations:** Instituto de Química, Universidade Federal do Rio de Janeiro, Avenida Athos da Silveira Ramos 149, Rio de Janeiro 21941-909, Brazil; diegobaiao20@ufrj.br (D.d.S.B.); davivieira@ufrj.br (D.V.T.d.S.)

**Keywords:** beetroot-food interventions, nitric oxide, betanin, polyphenols, antioxidant activity, clinical trials

## Abstract

The cardioprotective effects of dietary nitrate from beetroot in healthy and hypertensive individuals are undeniable and irrefutable. Nitrate and nitrate-derived nitrite are precursors for nitric oxide synthesis exhibiting an effect on cardiomyocytes and myocardial ischemia/reperfusion, improving endothelial function, reducing arterial stiffness and stimulating smooth muscle relaxation, decreasing systolic and diastolic blood pressures. Beetroot phytochemicals like betanin, saponins, polyphenols, and organic acids can resist simulated gastrointestinal digestion, raising the hypothesis that the cardioprotective effects of beetroots result from the combination of nitrate/nitrite and bioactive compounds that limit the generation of reactive oxygen species and modulate gene expression. Nitrate and phytochemical concentrations can be adjusted in beet formulations to fulfill requirements for acute or long-term supplementations, enhancing patient adherence to beet intervention. Based on in vitro, in vivo, and clinical trials, beet nitrate and its bioactive phytochemicals are promising as a novel supportive therapy to ameliorate cardiovascular diseases.

## 1. Introduction

Vegetables are important components of a balanced diet due to their constituents, comprising many bioactive compounds. These compounds, termed functional nutrients, provide benefits for the promotion and maintenance of human health [1,2]. Epidemiological studies have demonstrated that dietary nitrate (NO_3_^−^) from certain vegetables can provide a physiological substrate for the production of nitric oxide (NO) which, in turn, supports cardiovascular function, causes vasodilation, and decreases blood pressure [3,4,5,6].

Furthermore, secondary metabolites found in vegetables are involved in protective responses to different abiotic plant stresses [6]. In the last decade, systematic reviews and meta-analyses have demonstrated the potential health benefits of the dietary intake of plant polyphenols, mainly antioxidants, to decrease the risk of chronic and degenerative diseases [7,8,9]. It is estimated that at least 8,000 polyphenols have been described, considering natural, semi-synthetic, or synthetic compounds. Food matrices generally contain a complex mixture of those compounds, at variable concentrations, which may not yet have been well characterized [3].

Red beetroot (*Beta vulgaris* L. species) is a source of bioactive compounds, including dietary NO_3_^−^, betanin, antioxidant substances, and phenolic compounds (PCs), as well as a source of dietary fiber, minerals (potassium, sodium, iron, copper, magnesium, calcium, phosphorus, and zinc) and vitamins (retinol, ascorbic acid, and B-complex) [3,10]. However, dietary NO_3_^−^ supplementation from beetroot requires smart formulations, to provide convenient serving portions while also containing effective concentrations of NO_3_^−^ and bioactive compounds as a feasible alternative to the consumption of whole in natura vegetables.

Traditional beetroot formulations, such as the cooked vegetable or fresh juice, must be offered in large amounts to reach pharmacological NO_3_^−^ concentrations, making it difficult to convince individuals to adhere to certain proposed nutritional interventions [1,11,12].

Advances in beetroot formulations and regimen administration are discussed herein, addressing the features of designed formulations regarding nutritional composition, functional phytochemicals, antioxidant capability, and the potential to improve NO production, enhance cytoprotective and ensure positive effects on hemodynamic parameters in healthy individuals and in patients presenting risk factors for developing cardiovascular diseases.

## 2. Beetroot (*Beta vulgaris* L.) Formulations

*Beta vulgaris* belongs to the Betoideae subfamily, within the Amaranthaceae/Chenopodiaceae alliance. Although originated in Europe and North Africa, red beets are now naturalized in several countries worldwide. This species develops better in deep, looser, acid soils rich in organic matter and in mild (20 °C) to cold temperatures (10 °C). In cold weather, the beetroot plant goes through the reproductive stage of its cycle and the vegetable attains its best color, taste, and quality [13]. The appearance of oblong to heart-shaped leaves occurs in the vegetative phase, around the stem, that grows erect. Floral tassel emission occurs with the production of 2–3 mm lenticular seeds comprising glomeruli during the reproductive stage [14]. The root system is composed of main and smaller roots, with lateral branching. The taproot is dark purplish-red, globular to long in shape, and develops almost on the soil surface [14,15].

Abiotic conditions, such as atmospheric humidity, extreme temperatures, low and high, exposure time to sunlight, and brightness can affect NO_3_^−^ accumulation in vegetables, although some agricultural management aspects, such as farming systems, soil fertilization, nutrient availability, and herbicide use must be considered to achieve this crop’s growth and development [16].

Despite these factors, the consumption of a regular serving portion of beetroot in nature or minimally processed cannot reach the effective NO_3_^−^ concentration capable of producing the cardioprotective effects. A combination of concentrated fresh beetroot juice and beetroot chips in different amounts can adjust bioactive compound concentrations and result in attractive and convenient NO_3_^−^-rich beetroot products. Formulations must be maintained at appropriate serving portions [17], avoiding drawbacks in sensitive patients, where the ingestion of large volumes of concentrated fresh beetroot juice provoke gastric discomfort accompanied by nausea and vomiting, making it difficult to adhere to long-term interventions.

Several beetroot formulations have been designed and tested according to the group population to be supplemented, to produce convenient and attractive dietary NO_3_^−^ sources to stimulate NO production and promote beneficial health effects [11,12,18,19]. A dietary intake superior to 6.3 mmol is necessary to increase NO levels and blood pressure reductions in both healthy individuals and those presenting cardiovascular diseases (CVD)-risk factors.

All distinct beetroot formulations, presenting particular physicochemical characteristics and nutritional composition, have been rated in clinical trials, to evaluate their health effects on distinct populations. Beetroot formulated as fresh concentrated juice or fermented juice, bread, powder, chips or crunchy slices, gel, and cereal-bar have all been used to supplement healthy and/or unhealthy volunteers [1,3,20,21].

Beetroot juice is the original formulation for dietary NO_3_^−^ supplementation [3,11] and has been applied as a prime for most novel formulations proposed in recent years, according to the aim of the pre-clinical or clinical studies and to the volunteer population to be tested. Fresh and concentrated beetroot juice is produced from beets after mixing in a food centrifuge processor without adding water. In placebo-controlled studies, depleted NO_3_^−^ beetroot juice was obtained by the removal of NO_3_^−^ by anion-exchange chromatography using PuroliteA-520E resin. Depleted-NO_3_^−^ juice displays similar sensory characteristics and is indistinguishable in color, taste, appearance, and texture from NO_3_^−^-rich beetroot juice.

Fermented beetroot juice can be formulated by spontaneous lactic acid fermentation or by use of starter cultures, enriching betalain content in a probiotic product used to supplement healthy men [20,21,22,23,24]. Beetroot-enriched bread were obtained by replacing 50% of total dough weight with white or red beetroot before baking, developing a well-accepted product, which is already being marketed [25,26].

A beetroot gel was formulated to supplement athletes with dietary NO_3_^−^ during sports competitions. The beetroot gel was prepared to mix the fresh and concentrated beetroot juice and powder obtained by crashing beetroot chips. The chips were prepared from frozen and freeze-dried beet slices crushed in a portable blender to prepare beet powder. The gel was then prepared with a mixture of beetroot juice, beetroot powder, and carboxymethyl cellulose at a 90:17:3 ratio. A depleted-NO_3_^−^ beetroot gel was formulated by mixing depleted NO_3_^−^ beetroot juice and Fuji apple (*Malus pumila* species) puree, in substitution to beetroot chips [12].

Crunchy beetroot slices can be produced by means of microwave rotating vacuum drying in industrial facilities, resulting in a beet formulation retaining the taste, odor, and nutritious characteristics of their fresh equivalents [27]. Recently, a novel beetroot formulation, a beetroot cereal bar, a snack food, to be consumed between major meals, was designed as a chronic dietary NO_3_^−^ administration to individuals who present risk factors for developing cardiovascular diseases (1). Beetroot-cereal bars were produced from the compaction of concentrated fresh beetroot juice and cereals, such as oats, wheat, soybeans, corn, and rice. This beetroot-cereal bar formulation is rich in nutrients and packed into 60 g pieces measuring 10 cm × 3 cm × 1.5 cm [1,28,29]. Beetroot juice, brown sugar, corn syrup, and citric acid comprised the ligand phase, whereas beetroot powder obtained from crushed chips, and rolled oats, whole oats, rice flakes, and honey comprised the dry phase ingredients. Ligand phase ingredients were dispersed at 90 °C in a water bath, cooled and then mixed with the dry phase ingredients and the cereal-bar matrix was then baked and packed individually, to be consumed twice a day as a snack [1].

## 3. Nutritional Composition of Beetroot Formulations

Beetroot-cereal bars presented the highest contents of protein, lipids, total dietary fibers, total sugars, fructose, glucose, sucrose, NO_3_^−^ and saponin when compared to beetroot gel, chips, and juice, considering 100 g of each formulation (Table 1). Maltose at a concentration of 3.63 ± 0.19 mg/100 g was detected in the beetroot-cereal bars, which originated from the cereals added to the dry phase. Nitrite (NO_2_^−^) (<0.5 mmol/100 g) and lipids were present at <1 mmol/100 g. Beetroot chips presented the highest carbohydrate content (and energy) when compared to other beetroot formulations.

The beetroot-cereal bar and beetroot-gel designed for NO_3_^−^ supplementation included the binding phase ingredients or carboxymethyl cellulose, respectively, increasing carbohydrate content and energy but maintaining low lipid concentrations in both formulations. The beetroot cereal-bar and gel are considered low-fat foods, according to Brazilian legislation for solid foods [29], but low lipid contents did not compromise the sensorial characteristics of these beetroot intervention products, such as flavor, texture, color, and aroma.

Beetroot-cereal bar, gel, and chips can be classified as dietary fiber sources, since they contain over 3% (*w/w*) fibers, in accordance with the Brazilian legislation [29]. Beetroot-cereal bars presented the highest total dietary fibers due to the addition of cereals—oat and rice—that contribute not only to dietary fiber but also enhance protein content and phytochemical concentrations and diversity. As widely recognized, a high dietary fiber intake decreases blood cholesterol levels, by increasing bile acid production or short-chain fatty acid synthesis, and inactivates pathogenic bacteria, while stimulating the proliferation of benefic bacterial flora that, in turn, boosts the immune system and prevents and manages gastrointestinal tract infections.

The physicochemical characteristics of beetroot food interventions were considered to design the new formulations, since high water activity (a_w_) may promote undesirable modifications, such as non-enzymatic browning and crispness reduction, sensory attributes inherent to cereal bars and chips. In addition, moisture-rich food matrices can favor the growth of spoilage microorganisms, consequently decreasing product shelf-life [28]. The moisture percentage of beetroot-cereal bar and chips was maintained lower than 15%, except for the beetroot gel and juice, which presented higher moisture, as expected for pasty and liquid food products [29]. Beetroot formulations were processed under satisfactory hygienic conditions, stored at cold temperature, and adequately packed in accordance with Brazilian legislation for human food consumption, taking into account the time intervention period.

## 4. Bioactive Compounds in Beetroot Product Interventions

Insufficient clinical evidence concerning the efficacy and safety dosage of bioactive compounds found in fruits and vegetables makes it difficult to recommend the intake of these phytochemicals. If they are consumed in a balanced diet, putative health benefits include decreased risk for chronic, i.e., cardiovascular, diseases, even if the physiological targets and mechanism of action of several of these non-nutrients are still not fully understood [30]. Many bioactive compounds found in fresh vegetables and fruits display antioxidant activity against harmful reactive oxygen species, while others stimulate cellular defense mechanisms, enhancing stress responses, competing for active enzymes and receptor binding sites in subcellular structures, modulating the gene expression of proteins/enzymes capable of acting against oxi-degenerative processes that may occur in molecules and cellular structures [31].

### 4.1. NO_3_^−^ and NO_2_^−^

Beetroot juice is the most common NO_3_^−^ source used for supplementation, although its NO_3_^−^ concentration is lower when compared to other beetroot formulations [5,32].

Dietary NO_3_^−^ concentrations normalized to 100 g or 100 mL of the product was higher in beetroot-cereal bars (14.0 ± 0.05 mmol) when compared to beetroot gel (6.30 ± 0.01 mmol), chips (6.90 ± 0.02 mmol), and juice (4.10 ± 0.01 mmol). NO_2_^−^ contents ranged in low concentrations, from 0.10 ± 0.02 mmol to 0.20 ± 0.01, with no physiological significance (Table 1). Most beetroot formulations must be offered in large serving portions to reach effective NO_3_^−^ concentrations, taking into account the objectives of each intervention. Thus, a serving portion of 200 mL of beetroot juice, 100 g of beetroot gel, and chips can be used to supplement over 6.3 mmol of dietary NO_3_^−^/day. However, some gastrointestinal effects, as well as beeturia, may occur, impacting adherence to long-term supplementation [5,20,31,33]. On the other hand, the beetroot-cereal bar design provides an easy way to administer the beet-intervention product, in a convenient serving portion, as a healthy snack containing effective but higher dietary NO_3_^−^ dosages (≈6.3 mmol in 45 g of product) than beetroot juice and gel, previously used to treat individuals at risk of developing CVD. Considered a snack, beetroot-cereal bars can be administered between meals, facilitating adhesion to NO_3_^−^ nutritional interventions. Due to the mixture of fresh juice and powder, NO_3_^−^ amounts can be adjusted and the beetroot-cereal bar can be used for both acute or chronic NO_3_^−^ supplementations, presenting beneficial cardiovascular system effects for both healthy and patient populations.

### 4.2. Saponins

Few studies report the saponin content of beetroot products, but it is known that saponin content and types may vary according to the plant cultivar and food matrix processing [12]. Saponin contents in beetroot food interventions ranged from 2599 ± 1.27 to 8648 ± 1.85 mg/100 g, and the cereal bar was verified as the richest source (Table 2). Interestingly, in soybean, considered the main dietary saponin source, contents found in germs, cotyledons, and soy molasses varied from 935 ± 50.7 to 6583 ± 250.5 mg/100 g, lower than in beetroot [34]. Beetroot intervention products should be considered adequate for dietary saponin supplementation and may eventually replace soybean.

Several beneficial bioactivities are attributed to isoprenoid or terpenoid compounds, where an aglycone is attached by a covalent bond to one or two sugar chains, forming a mono- or di-desmoside. Furthermore, oleanoic acids, betavulgarosides II, III, and IV, found in *Beta vulgaris* L. roots have been shown to promote hypoglycemic effects in rats. [35].

### 4.3. Organic Acids (OAs)

Beetroots are rich in OAs, similar to most plants, where these acids are used to cope with nutrient deficiencies, metal detoxification, and tolerance, and pathogens, as well as endophytic and symbiotic-microbe interactions operating at the root-soil interface [36]. Humans can also benefit from the ingestion of these compounds.

Beetroot-cereal bars present the highest total OA content (9.19 ± 0.71 mg/g) compared to chips (5.34 ± 0.35 mg/g), gel (4.17 ± 0.35 mg/g), and juice (2.84 ± 0.7 mg/g) (Table 2). Six distinct OAs including citric, ascorbic, malic, fumaric, succinic, and oxalic acids have been quantified in the beetroot-cereal bar, whereas succinic acid and oxalic acid have been found only in beetroot-cereal bars, both derived from the cereals added during bar formulation, while citric acid, ascorbic acid, malic acid, and fumaric acid are found in beets and present in all beet-derivatives. Malic acid and citric acid are the most abundant in beet formulations [1] (Table 2). The overall OA content found in some beetroot product interventions is close to those found in the most dense-dietary sources of OAs, such as kefir (≈12.0 mg/mL) and milk (≈5.0 mg/mL) [37].

In roots, OAs are present as partially neutralized potassium (K^+^) salts, such as those formed by citrate, malate, and, less efficiently, by oxalate, and their contents can be influenced by soil characteristics, temperature and precipitation regimes, conventional or organic farming systems and post-harvest processing (fresh, cooked, juice, or chips) [38].

Phosphoric acid and citric acid are predominant in beetroot juice, followed by oxalic acid and malic acid. Subsequently, shikimic acid, the precursor for the synthesis of aromatic amino acids such as phenylalanine, tyrosine and tryptophan, and betalains, are detected in high concentrations in organic and conventional farming beets, while citric acid, malic acid, and fumaric acid are also observed, but at lower concentrations [39]. Malic acid is present at the highest concentrations in beetroot formulations, including juice, chips, powder, and cooked vegetables, followed by citric acid and ascorbic acid [18].

Some OAs are involved in the beneficial effect promoted by certain foods against oxidative stress, aiding in chronic and degenerative conditions, including cardiovascular diseases [40].

Malic acid is a putative adjuvant in the conservative treatment of calcium (Ca^2+^) renal stone disease, due to its potential ability to complex with Ca^2+^ in urine, preventing the formation of Ca^2+^ oxalate (CaOx), the main kidney stone component. Malic alkalizing effects increase citrate excretion, improving hypocitraturia [41,42,43,44].

Citric acid acts as a synergistic antioxidant alongside other compounds and has been pointed out as a chelating agent, protecting molecules from metal-catalyzed oxidation [45,46]. Like malic acid, the ingestion of foods rich in citric acid can be an alternative for the treatment of hypocitraturia, reducing predisposition to renal stone formation [35,47].

Ascorbic acid, known as vitamin C, is a potent antioxidant also present in beetroot formulations (Table 2). Ascorbic acid contents found in cereal bars (1.55 ± 0.21 mg/g) and chips (0.53 ± 0.04 mg/g) are higher than in citrus fruits (0.53 mg/g), i.e., orange and lemon, which are considered good sources of vitamin C but present similar amounts to those reported in beetroot chips [48]. Ascorbic acid is a powerful antioxidant, able to donate a hydrogen atom, generating the ascorbyl-free radical to protect biomolecules from damage caused by oxidative compounds generated in cell metabolism or following exposure to xenobiotic compounds [49]. Vitamin C functions as a cofactor for monooxygenase and dioxygenase enzymes involved in the degradation or detoxification of toxins and pollutants [50].

Ascorbic acid can also regulate collagen synthesis in blood vessels and, alongside NO_3_^−^ and NO_2_^−^, improve cardiovascular function. In addition, it also plays a role as a cofactor for pro-collagen enzymes, such as lysyl and prolyl hydroxylases, generating substrates for collagen biosynthesis [51,52,53,54], stabilizing the collagen triple helix, and the formation of intermolecular collagen crosslinks [55,56,57]. Finally, ascorbic acid also stimulates collagen mRNA production in fibroblasts [54,58,59] and is a cofactor for carnitine biosynthesis, modulating the entry of long-chain fatty Acyl-CoA esters into mitochondria for β-oxidation [60].

Fumaric acid, (E)-butenedioic acid, present in beetroot formulations cereal bars at 0.81 ± 0.1 mg/g, in chips at 0.63 ± 0.1 mg/g, gel at 0.41 ± 0.2 mg/g, and juice at 0.18 ± 0.1 mg/g (Table 2), is a dicarboxylic acid which has emerged as an adjuvant to autoimmune disease therapies, such as multiple sclerosis, psoriasis, oxidative stress, and Parkinson’s disease [61,62,63,64,65,66].

Succinic acid (butanedioic acid) was detected at 0.51 ± 0.01 mg/g in beetroot cereal bars (Table 2). This acid is involved in angiogenesis via the vascular endothelial growth factor, epidermal growth factor receptor, platelet-derived growth factor, and glucose transporter 1, while also participating in the crossing to other metabolic pathways, such as the tricarboxylic acid cycle and the respiratory chain [67,68,69,70]. Another important succinate role is the activation of succinate-receptor 1 (SUCNR1) signaling, promoting the generation of endothelial NO and prostaglandin E2 (PGE2), and the synthesis and release of renin, supporting blood pressure regulation by the renin-angiotensin system [71,72]. Succinate is also involved in mitochondrial integrity by maintaining the ubiquinone (CoQH2) pool and inhibiting mitochondrial lipid peroxidation [73,74,75]. Therefore, succinic acid could support the vascular effects of beet NO_3_^−^.

### 4.4. Phenolic Compounds (PCs)

A large body of epidemiological evidence and meta-analyses has demonstrated that dietary PCs [76] can aid in the prevention of chronic conditions [77] such as neurodegenerative diseases [78,79], cancers [80,81], inflammation, diabetes, and obesity [82,83,84], and cardiovascular diseases [84].

To the best of our knowledge, human intervention trials assessing the direct effect of each beetroot compound, such as betagarin, betavulgarin, flavonoids, vanillic, *p*-coumaric, and syringic phenolic acids, are not yet widely available. When tested in cell cultures and animals, some of these compounds have shown antibacterial, anti-inflammatory, antioxidant, anti-tumoral, and protective effects against reperfusion ischemia injury [85,86,87,88].

Beetroot is a dietary source of PCs, although their concentrations vary according to the plant part, high in plant skin, and less concentrated in the crown and flesh [89,90]. PCs identified in beetroot juices obtained from organic and conventional cultivars and beet varieties include ferulic, caffeic, gallic, *p*-coumaric, chlorogenic, *p*-hydroxybenzoic, syringic and vanillic acids, quercetin, and myricetin [37,91].

Gallic acid, 3.4-dihydroxybenzoic acid, syringic acid, caffeic acid, chlorogenic acid, and ferulic acid have been detected in all beetroot formulations. Beetroot-cereal bars showed the highest PC content, 147.73 ± 3.3 mg/100 g, also displaying greater diversity when compared to beetroot chips (42.62 ± 1.39 mg/100 g), gel (25.45 ± 1.42 mg/100 g), and juice (18.00 ± 0.155 mg/100 mL) (Table 2). PCs identified and quantified in beetroot-cereal bars also include *p*-coumaric acid, rosmarinic acid, syringic acid, and vanillic acid (Table 2).

Gallic acid is described as the most abundant beetroot polyphenol [37,91,92]. Beetroot-cereal presented the highest content of gallic acid (60.50 ± 1.76 mg/g), followed by chips (22.49 ± 1.17 mg/g), gel (8.81 ± 0.15 mg/g) and juice (4.10 ± 0.06 mg/g) (Table 2). Gallic acid has had various biological functions evidenced in humans, including control of glucose metabolism and ameliorating inflammatory and oxidative stress-related complications [93,94,95,96,97,98,99,100,101,102,103,104]. Ferulic acid, a hydroxycinnamic acid derivative, has been identified in all beetroot food interventions (Table 2). The phenolic nucleus and the extended carboxylic chain in the ferulic acid molecule form a resonance-stabilized phenoxy radical, explaining its high antioxidant potential [105,106,107,108,109]. Ferulic acid had a cardiometabolic effect, by attenuating inflammation, oxidative stress [110,111,112,113,114,115,116], and other risk factors for cardiovascular disease [107,117,118,119,120,121,122].

Beetroot products present caffeic acid concentrations of over 3.17 ± 0.45 mg/g (Table 2).

Caffeic acid displays antioxidant and anticancer activities [123,124,125,126], protecting several organs as lungs [127,128], mouth [129,130], liver [131,132], and colon [133,134,135]. Caffeic acid can impair macromolecule damage, balancing oxidative stress conditions, and its high antioxidant activity can be ascribed to the hydroxyl groups and the ortho-dihydroxyl group in the caffeic acid molecule [136]. Caffeic acid is more effective than *p*-coumaric acid and ferulic acid in inhibiting copper-mediated oxidative modifications of human LDL, and consequently, in reducing the pathogenesis of atherosclerosis [137,138,139].

Chlorogenic acid is a class of compounds formed by hydroxyl cinnamic esters with quinine acid [140,141]. Beetroot-product interventions have reported chlorogenic acid concentrations ranging from 5.94 ± 0.033 mg/100 g in cereal bars to 2.90 ± 0.003 mg/100 g in juice (Table 2).

A growing body of evidence supports the therapeutic effects of chlorogenic acid, including antioxidant activities, hepato, and cardioprotective properties, anti-inflammatory, anti-obesogenic and anti-hypertensive abilities, its ability to influence glucose homeostasis, and a neuroprotective role [142,143,144]. The antioxidant and anti-inflammatory effects exerted by chlorogenic acid are mediated through the Nrf2-ARE pathway, where the transcriptional factor NE-F2-related factor-2 binds to antioxidant responsiveness elements and promotes the up-regulation of anti-oxidative genes, such as heme oxygenase-1 (HO-1), NAD(P)H dehydrogenase quinone 1 (NQO1), glutamate-cysteine ligase (through its catalytic subunit–GCLC) [145,146,147]. Regarding vascular function, the intake of purified chlorogenic acid inhibits aspartate and alanine aminotransferases, lipid peroxidation, and improves continuous post-ischemic dilatation-mediated flow in healthy individuals, who presented sustained vascular function improvement [148,149,150].

### 4.5. Betalains

**Figure 1:**
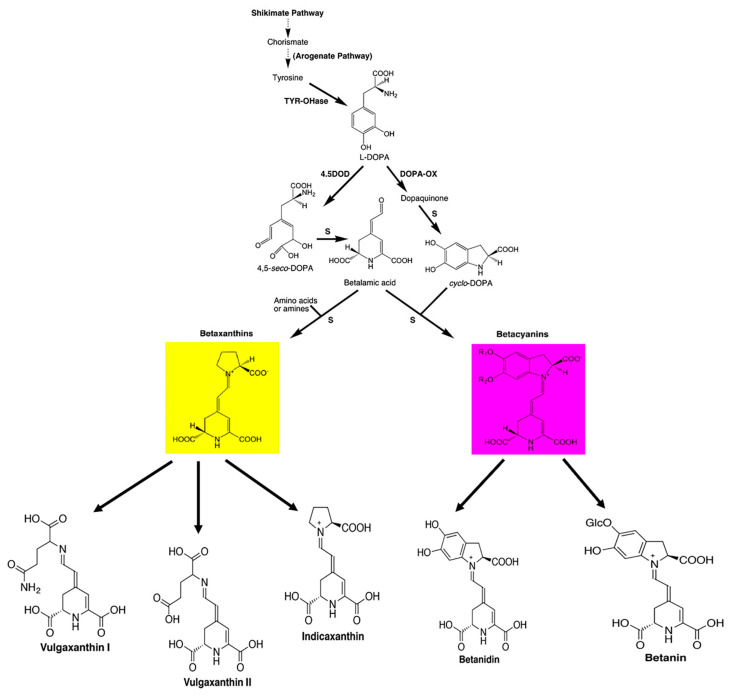
Biosynthesis pathway and general structures of betalains (reproduced from [161]).

Betalains are aromatic indole derivatives, comprising nitrogen-containing and water-soluble pigments that confer beetroot color. These pigments are widely distributed in plant tissues and organs of members belonging to the *Caryophyllales* order. Betalains are synthetized from tyrosine via the shikimate pathway [151,152,153,154,155,156]. Betalains are divided into two subclasses according to their color: the yellow pigments betaxanthins mainly represented by vulgaxanthin I, II, and indicaxanthin, exhibiting maximum absorption from 460 to 480 nm; and betacyanin red pigments mainly represented by betanin, exhibiting maximum absorption from 535 to 540 nm [157,158,159,160] (Figure 1).

Red beetroot is an excellent source of betanin (75–95%) but it also contains lower concentrations of isobetanin, betanidin, and betaxanthin [162,163]. Betanin content in red beet may be affected by farming conditions, including soil fertilization, moisture, post-harvest storage conditions, and, mainly, exposure to light and high temperatures [89,90,164].

Considering the betanin content found in beetroots and prospecting the amount in different beetroot formulations, beet chips would show the highest content (1274 mg/g) followed by juice, gel, and cereal bars [165] (Table 2).

In the food industry, betanin obtained from beetroot is used in sorbets, dairy derivatives like yogurts and ice creams, as well as meats (i.e., sausage), since betanin display good stability in a wide pH range (pH 3–7). The use of betanin as a natural red-violet dye for food is regulated by the Food and Drug Administration (FDA) and European Food Safety Authorities, under E-number E162 [166,167]. Betanin can also be considered a natural food preservative and alternative to synthetic antioxidants (i.e., BHA and BHT), due to its ability to prevent lipid peroxidation [165,168].

The exact mechanisms of betanin absorption, metabolic breakdown, and route excretion in humans have not yet been completely elucidated, and identification of chemical intermediates, such as glucuronides, sulfates, or conjugates of methylated betalain, in plasma and urine is still scarce. It is known that the bioavailability of betanin can be influenced by the source matrix (i.e., different food sources or forms of preparation) and by human interindividual variability such as genetics, sex, age, and health conditions, which alter its absorption and excretion profile [24,27,169,170,171].

Betanin stability and antioxidant ability have been evaluated in assays mimicking in vitro human digestion and ex vivo colonic fermentation [165]. Over half of the original betanin content is preserved after oral, gastric, and small intestine digestion, as observed in vitro simulation. No betanin was recovered from the ex vivo colon fermentation assay. The betanin chemical structure was preserved during simulated gastrointestinal digestion, as well as its antioxidant activity, confirmed by different antioxidant assays. The ability of betanin to inhibit the OH-radical within the total antioxidant potential (TAP) and its reductive ability to alter the ferric ion of the tripyridyltriazine complex (Fe^3+^-TPTZ) to the ferrous ion (Fe^2+^-TPTZ) was demonstrated in the ferric reducing ability of plasma (FRAP), as well as in the reduction of the 2,2′-azino-bis(3-ethylbenzothiazoline-6-sulfonic acid (ABTS^+^) radical in the trolox equivalent antioxidant capacity (TEAC) and oxygen radical antioxidant capacity (ORAC) assays [165]. In agreement with these findings, betanin absorption through epithelial cell membranes occurred with no chemical transformation in a trans-epithelial transport assessment carried out with Caco-2 cells, [171].

Human betanin bioavailability is low as 2.7% of total oral intake is excreted in urine and feces [165,172,173,174]. Betanin reaches a maximum plasma concentration after ≈3 h and is no longer detected after 12 h of ingestion [169]. Absorbed betanin is excreted primarily by urine, and some individuals can present reddish urine (beeturia) following oral administration [175], while renal excretion is lower than 3% of the administered dose [173]. A very small part of administered betanin (≤1%) through the ingestion of beet juice was excreted in the urine of volunteers after 2–7.5 h mainly as isobetanin, suggesting the occurrence of betanin isomerization due to the temperature of the human organism [175,176]. In addition, other trials have shown that about 90% of the betanin and isobetanin ingested are rapidly excreted as an unchanged structure from 0 to 4 h after administration, indicating that a part is quickly absorbed, while excretion between 8–24 h occurs predominantly in its aglycone form (betanidin and isobetanidin) [27]. The plasma bioavailability of betanin has shown significant variability in different clinical trials, perhaps due to the aforementioned biological variability of each individual and differences in administered doses, although, the low detection of betanin in plasma is a common finding. In another study, betanin was not detected in plasma at any time point post-ingestion of 250 mL of beetroot juice or 300 g of whole beetroot, containing near 194 and 66 mg of betanin respectively [177]. Minimal amounts of betanin in plasma (< 1 µg), 3, 8, and 24 h after supplementing male patients with extracts containing 16 and 35 mg of betanin and after 2 weeks of supplementation (<3 µg) have been reported [178]. Regular consumption for long periods (between 1 and 6 weeks) of beetroot products seems to be the solution to overcome low betanin levels in biological fluids by promoting stabilization of the systemic levels, where betanin and their deglucosylated, decarboxylated and dehydrogenated metabolites are consistently described [24]. The free radical scavenging activity of betanin, due to its ability to donate electrons and hydrogen, relies on the cyclic amine present in its structure, resembling ethoxyquin, a strong antioxidant, as well as hydroxyl groups (-OH), which are excellent hydrogen donors [176]. Due to its ability to remove reactive oxygen species (ROS), betanin prevents oxidative damage to lipid macromolecules and DNA, reversing tissue damage [179,180,181].

**Figure 2:**
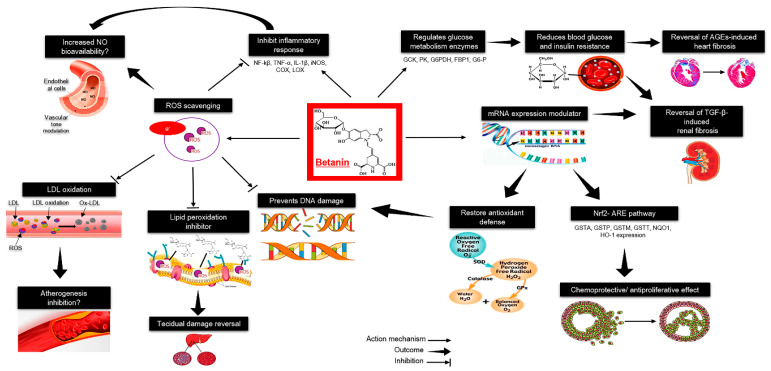
Health effects of betanin: A summary of molecular and metabolic targets of betanin reported in cell cultures and animal models. AGEs, advanced glycation end products; C, carbon; COX, cyclooxygenase; DNA, deoxyribonucleic acid; FBP1, fructose-bisphosphatase 1; G6-P, glucose 6-phosphate; G6PDH, glucose-6-phosphate dehydrogenase; GCK, glucokinase; GPx, glutathione peroxidase; GSTA, glutathione S-transferases A; GSTM, glutathione S-transferases M; GSTP, glutathione S-transferases P; GSTT, glutathione S-transferases T; H, hydrogen; H_2_O_2_, hydrogen peroxide; IL-1β, interleukin 1 beta; HO-1, heme oxygenase-1; iNOS, inducible nitric oxide synthase; LDL, low-density lipoprotein; LOX; lipoxygenase; mRNA, messenger ribonucleic acid; N, nitrogen; NF-_Κ_β, nuclear factor kappa beta; NQO1, quinone dehydrogenase 1; NO, nitric oxide; Nrf2-ARE, nuclear factor erythroid 2-antioxidant responsive element; O, oxygen; O2^•−^, superoxide anion; OH, hydroxyl radical; Ox-LDL, oxidized low-density lipoprotein; PK, pyruvate kinase; ROS, reactive oxygen species; SOD, superoxide dismutase; TGF-β, transforming growth factor beta; TNF-α, tumor necrosis factor alpha.

In vascular tissue, betanin antiradical activity maintains endothelial function and reduces the atherogenesis process (Figure 2). In addition, betanin can modulate redox-mediated signal transduction pathways involved in inflammation responses in endothelial cells by inhibiting the intercellular cell adhesion molecule-1 (ICAM-1), resulting in antiproliferative effects in human tumoral cells [182,183].

Since excessive ROS are removed by betanin, NF-κB activation, and cytokine expression down-regulation are noted [184]. Betanin also regulates liver glucose metabolism-related enzymes in diabetes type II, such as those involved in the glycolytic pathways, like glucokinase, glucose-6-phosphatase, pyruvate kinase, in the pentose phosphate pathway, i.e., glucose-6-phosphate dehydrogenase, and in gluconeogenesis, like fructose-1,6-bisphosphatase [185]. Chronic hyperglycemia promotes tissue fibrosis mediated by advanced glycation end products (AGEs) and transforming growth factor-beta (TGF-β). The antidiabetic role of betanin has been proven to revert hyperglycemia, hyperinsulinemia, insulin resistance, and glycation products in rats induced to experimental diabetes by high-fructose intake, orstreptozotocin-nicotinamide, or high-fat hypercaloric diet [186,187,188].

Therefore, the effects of betanin on inflammation, oxidative stress, and diabetes in rodent models are well documented, these findings have not yet been confirmed in humans.

## 5. Beetroot Product Interventions Increase Nitric Oxide Production and Promote Health Benefits

Vegetables are important health-promoting foods in a balanced diet, due to the bioactivities of their phytochemicals [189,190]. It is widely recognized that dietary NO_3_^−^ from beetroot and green leafy vegetables may provide a physiological substrate for the generation of NO and other bioactive nitrogen oxides, leading to vasodilation and consequent improvement in cardiovascular function [191].

Dietary NO_3_^−^ is well absorbed in the upper gastrointestinal tract. About 25% of dietary NO_3_^−^ is captured by the salivary glands, where it is reduced to NO_2_^−^ by commensal bacteria that express and secret NO_3_^−^-reductase enzyme in saliva [6,191]. The metabolic activity of the hundreds of commensal bacteria species belonging to the *Granulicatella*, *Actinomyces*, *Veillonella*, *Prevotella*, *Neisseria*, *Haemophilus*, and *Rothia* genera that live on the tongue can directly influence the NO_3_^−^ to NO metabolism. Individuals with a higher abundance of NO_3_^−^-reducing bacteria were able to generate more salivary NO_2_^−^ and, consequently, NO at a faster rate following the ingestion of dietary NO_3_^−^ [192]. In contrast, the enzymatic activity of bacteria in the mouth and conversion of NO_3_^−^ to NO_2_^−^ may be disrupted by antibiotic use or mouth rinsing with an anti-bacterial mouthwash. Oral nitrate-reducing microbiota are beneficial to the host and participate in the control of cardiovascular NO homeostasis [6,192,193].

After the conversion of dietary NO_3_^−^ to NO_2_^−^ in the oral cavity, the NO_2_^−^ in the saliva is swallowed and reaches the stomach, where NO_2_^−^ is non-enzymatically decomposed into NO and other bioactive nitrogen oxides in this acidic environment, by vitamin C or polyphenols. In addition to dietary sources, NO_3_^−^ and NO_2_^−^ can be endogenously originated from NO synthetized by the three isoforms of the nitric oxide synthase (NOS), family from the amino acid L-arginine and O_2_, namely the neuronal (nNOS or NOS-I) and endothelial (eNOS or NOS-III) isoforms, both constitutive and dependent on Ca^2+^-calmodulin, and the inducible isoform (iNOS or NOS-II). In addition, L-arginine is metabolized by arginase to L-ornithine and urea to eliminate excess nitrogenous compounds [193].

NO is a low molecular weight compound (30.01 g/mol) with a short-life (from 5 to 10 s) produced in gas form, containing 11 electrons in its valence shell with an unpaired electron. This radical character confers high reactivity to this compound, since it rapidly oxidizes to NO_2_^−^ and NO_3_^−^. NO displays an affinity for lipophilic environments and accumulates in the lipid milieu, such as cell membranes and lipoproteins [193]. In human physiology, NO can exert antioxidant functions and is considered a secondary messenger, acting on the vascular endothelium, central and peripheral neurons, and immune system, inhibiting platelet activation, adhesion, and aggregation, modulating vascular tone, and improving human skeletal muscle function [5,27,194,195].

**Figure 3:**
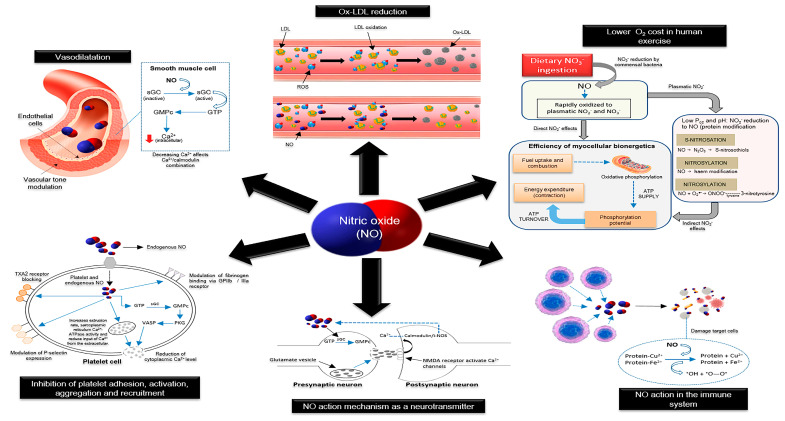
The physiological role of nitric oxide in smooth muscle tissue, maintenance of vascular tone, synaptic transmission, cellular defense, hemostatic-thrombotic balance, and mitochondrial function. ATP, adenosine triphosphate; ATPase, adenosine triphosphatase; Ca^2+^, calcium; Cu^2+^, copper; Fe^2+^, ferrous iron; GMPc, guanosine monophosphate cyclic; GPIIb, glycoprotein IIb; GPIIIa, glycoprotein IIIa; GTP, guanosine-5′-triphosphate; iNOS, inducible nitric oxide synthase; LDL, low-density lipoprotein; N2O3, dinitrogen trioxide; NMDA, N-methyl-D-aspartate; NO, nitric oxide; NO_2_^−^, nitrite; NO_3_^−^, nitrate; O_2_, oxygen; ONOO^−^, peroxynitrite; Ox-LDL, oxidized low-density lipoprotein; PKG, protein kinase G; P_O2_, pressure of oxygen; ROS, reactive oxygen species; sGC, soluble guanylate cyclase; TXA2, thromboxane A2; VASP, vasodilator-stimulated phosphoprotein.

Multiple pathways are used by NO to promote these actions, which depend on the cell tissue and the amount of produced NO (Figure 3). As mentioned previously, NO’s free-radical scavenging ability reduces ROS, promoting cardioprotective effects on the atherosclerotic process by preventing LDL cholesterol oxidation, and reducing RNO production rates [196].

In immune cells, NO is produced as part of the inflammatory response by macrophages and other immune system cells, which express the inducible isoform type II NO synthase. The formed NO reacts with the superoxide anion (O2^•−^), generating peroxynitrite (ONOO^−^), which, in turn, causes lethal damage to pathogens or tumoral cells by attacking copper and iron-metalloproteins [5,27,192].

NO formed by the neuronal NO synthase (nNOS) acts as a neurotransmitter in the central and peripheral nervous systems, mediating synapse plasticity in nerve impulse transmission and favoring the secretion of neurotransmitters or hormones in neuronal junctions. The nervous impulse transmission occurs when glutamate, the main excitatory neurotransmitter, diffuses from the presynaptic terminal to bind to the N-methyl-D-aspartate type (NMDA) receptors at the postsynaptic terminal. NMDA receptors are coupled to Ca^2+^ ion channels and their activation by glutamate allows the flow of Ca^2+^ into the postsynaptic terminal. Ca^2+^ associates with calmodulin and activates nNOS, promoting the formation of NO. NO may diffuse to the presynaptic terminal and stimulating the generation of cyclic guanosine monophosphate (cGMP) from guanosine-5′-triphosphate (GTP) catalyzed by the soluble guanylate cyclase (sGC), cGMP then activates protein kinases triggering phosphorylation of target enzymes, activating or inhibiting them [194]. However, the predominant mechanism that mediates the effects of NO signaling in the nervous system involves post-translational modification of thiol nitrosylation of Cys residues, termed S-nitrosylation, Tyr nitration, termed 3-nitrotyrosination (NO_2_^−^Tyr via ONOO^−^ formation), and PKG-dependent phosphorylation of Ser residues of the target proteins [197].

Both endothelium- and platelet-derived NO prevent platelet aggregation and fibrin formation, inhibiting the spread of thrombi generation [196]. NO exerts its inhibitory action by reducing cytoplasmic Ca^2+^ through increasing Ca^2+^ extrusion rates and sarcoplasmic reticulum Ca^2+^-ATPase and decreased Ca^2+^ input from the extracellular medium. NO promotes phosphorylation of thromboxane-2 receptor and down-regulates P-selectin expression, preventing platelet activation and adhesion [198]. In addition, NO modulates fibrinogen binding via the glycoprotein IIb and IIIa (GPIIb/IIIa) receptor, increasing the dissociation constant of this receptor by fibrinogen, reducing the total number of GPIIb/IIIa receptors on the platelet surface, resulting in unfavorable conditions for platelet aggregation. Furthermore, NO stimulates tyrosine nitrosylation in the ONOO^−^ pathway, thereby inhibiting thromboxane-2 synthesis [199].

NO regulates vascular tone by diffusing across endothelial cells, reaching vascular smooth muscle cells and, through sGC, activates the sarcoplasmic Ca^2+^ pump, decreasing intracellular Ca^2+^ and promoting vasodilation as a result of diminished vascular tone [200].

Under low O_2_ levels and pH, any member of the NO_2_^−^ reductase class enzymes, including xanthine, aldehyde oxidases, aldehyde dehydrogenase type 2, carbonic anhydrase, or deoxyhemoglobin, can reduce NO_2_^−^ to NO [201]. The NO generated alongside NO_2_^−^ from the dietary-NO_3_^−^ conversion improves oxidative phosphorylation efficiency, evidenced by an increased P/O ratio, indicating no uncoupling mechanisms, such as proton leaks towards ATP synthesis and turnover, improving ATP supply to skeletal muscle [27].

Several studies report beneficial effects of dietary NO_3_^−^ in the stimulation of NO production and biochemical, hemodynamic, and vascular parameters following the intake of doses ranging from 6.3 to 22.0 mmol. Different beetroot product interventions have been formulated with distinct nutritional compositions and tested to achieve their claimed health effects (Table 3) [12,19,33,202].

However, to obtain the maximum cardioprotective effect of NO_3_^−^ intake, the dosage, supplementation regimen and the health status of the assessed individuals must be considered. Minimal or no hemodynamic and vascular beneficial effects in healthy individuals have been observed following acute NO_3_^−^ administration from 1 to 7 days. An intake of 7.0 mmol of NO_3_^−^ in 140 mL of beetroot juice by 27 treated-hypertensive volunteers for 7 days resulted in increased NO synthesis, as assessed by plasmatic, urinary, and salivary NO_3_^−^ and NO_2_^−^, but no differences in home blood pressure (BP) and 24 h ambulatory systolic (SBP) and diastolic blood pressure (DBP) [203]. A supply of 9.92 mmol of NO_3_^−^ in 100 g of beetroot gel to 25 healthy and physically active runners for 1 week promoted increases in urinary NO_3_^−^, creatinine, and NO_2_^−^ after 90 min of beetroot ingestion and after exercise. However, urinary levels of nitrous compounds were not related to changes in oxygen volume (VO_2peak_), time to fatigue during treadmill running, respiratory quotient, SBP, and DBP [19].

Acute ingestion, of higher doses, of ≈13.0 mmol of NO_3_^−^ in 200 mL of beetroot juice, by 14 non-hypertensive obese males increased NO_3_^−^ + NO_2_^−^ (NOx) plasma concentrations (from 9.9 ± 8.4 μM to 47.0 ± 16.9 μM), which remained elevated until 1h post-intervention (54.7 ± 10.1 μM), while no changes in 24 h ambulatory SBP and DBP were detected [209]. Minimal effects were observed in 15 healthy volunteers treated by 7.3 mmol of NO_3_^−^ on brachial SBP, not sustained over 24 h, and carotid to femoral pulse wave velocity (_cf_PWV) [213]. Regardless of the use of higher doses of dietary NO_3_^−^ and a proved improvement in NO synthesis, these aforementioned results indicate a critical role of vascular impairment caused by some chronic non-communicable diseases such as hypertension, dyslipidemia, obesity, and aging, impairing NO effects in target tissues [218]. Furthermore, NO_3_^−^ supplementation benefits on physical performance have been suggested as more meaningful in healthy, but non physically active, individuals, rather than active ones. Physiological adaptations of endurance training may stimulate the expression and activity of the NOS enzyme through the endogenous pathway (via L-arginine/NO), increasing NO bioavailability. Due to the activation of the NO endogenous biosynthesis, the dependency of NO bioavailability derived from dietary NO_3_^−^ supplementation seems to be reduced [19].

However, in individuals presenting one or more risk factors for the development of cardiovascular diseases, the reversal of endothelial dysfunction evaluated by decreased large-artery stiffness and BP is achieved following the intake of up to 6.0 mmol of NO_3_^−^ if long-term supplementation is performed [5,204,218]. Three weeks administration of 6.45 mmol of NO_3_^−^ in 70 mL beetroot juice to 24 older and overweight volunteers promoted an increase in NO synthesis, estimated by urinary and salivary NO_3_^−^ and NO_2_^−^, resulting in SBP decreases of up to 7.3 mm Hg [202]. The intake of 250 mL of beetroot juice containing 6.4 mmol of NO_3_^−^ by 34 drug-naive hypertensive patients for 4 weeks increased NO synthesis and cGMP levels, accompanied by decreases in arterial stiffness and a ≈20% improvement in endothelial function proven by decreases in 24 h ambulatory and home BPs [29]. The intake of 60 g of beetroot-cereal bars containing 9.57 mmol of NO_3_^−^ for 3 weeks by five patients presenting at least three risk factors for the development of CVD promoted increases in the NO synthesis and improvements in cutaneous microvascular conductance peak decreases in arterial stiffness (through assessments concerning the augmentation index—AIx, aortic pulse pressure—_ao_PP, and PWV index) and decreases in SBP and DBP [208].

A systematic review and meta-analysis study of randomized controlled trials demonstrated that inorganic NO_3_^−^ and beetroot supplementation can improve endothelial function. Beetroot juice intake provoked decreases in SBP (−3.55 mm Hg; 95% CI: −4.55, −2.54 mm Hg) and DBP (−1.32 mm Hg; 95% CI: −1.97, −0.68 mm Hg) [218] associated with the ingestion of beetroot juice and supplementation periods of over 14 days. Inorganic NO_3_^−^ effects on endothelial function were associated with dose, age, body mass index (BMI), and previous SBP.

Chronic beetroot juice ingestion improved vascular performance, evaluated by flow-mediated dilation (FMD) and endothelium functional effects according to the administered NO_3_^−^ dose (β = 0.04, SE = 0.01, *p* < 0.001), age (β = −0.01, SE = 0.004, *p* = 0.02) and BMI (β = −0.04, SE = 0.02, *p* = 0.05). A critical review of experimental data confirmed that NO_3_^−^ is a positive vascular endothelium effector, promoting vasodilatation and reducing blood pressure in both normal and hypertensive individuals. Beneficial effects were shown to be dependent on both NO_3_^−^ dosage and continued intervention. Furthermore, beetroot product interventions designed to fulfill healthy effects are well tolerated even during long-term administration of super-concentrated beet products.

The data compiled herein shows that the cardioprotective effects of beetroot NO_3_^−^ reported by several independent clinical trials performed worldwide are incontestable, increasing the importance of considering the individual susceptibilities and health status of each organism. Successful NO_3_^−^ supplementation depends on the dosage and supplementation regimen, as well as the dietary source of NO_3_^−^. Impaired NO status can result from unsatisfactory production and/or reduced bioavailability, explaining why high NO_3_^−^ supplementation dosages are necessary to fully overcome deficient endogenous NO synthesis or force the absorption rates. Therefore, beetroot consumption has emerged as an alternative, convenient and attractive way to obtain the cardioprotective NO_3_^−^ effects in healthy individuals presenting risk factors for CVD risk, due to the higher concentration of NO_3_^−^ per vegetable weight. However, the large serving portion of beetroot formulations necessary to achieve the effective dose of dietary NO_3_^−^ can cause gastrointestinal adverse effects, limiting adherence to long term treatments. Furthermore, the aforementioned studies suggest that frequent daily doses of dietary NO_3_^−^ for long periods would be necessary to result in beneficial effects on blood pressure and endothelial function and should be recommended to populations with compromised vascular responsiveness.

## 6. Bioactive Beetroot Compounds—NO_3_^−^ and Betanin—Modulate the Transcription of Genes Responsible for Regulating Redox Imbalance in a Rodent Model

The cellular and systemic improvements observed after dietary NO_3_^−^ intervention may be due to up- and down-gene expression in endothelial function regulation and platelet and macrophage recruitment and vasodilation, while also reducing imbalances in the redox state of the cardiovascular system, associated with mRNA inhibition of endogenous ROS generators, as well as NADPH oxidases. Meanwhile, activations of GPx, CAT, and SOD gene expressions are also noted, increasing the availability of scavenging enzymatic effectors [188].

Transcriptional patterns in aged mice whole thoracic aortas after chronic NaNO_3_^−^ supplementation highlight changes in the expression of genes encoding the calcium-signaling pathway, as well as in detoxification and antioxidant defenses. As a long-term effector, NO_3_^−^ promoted up-regulation of genes encoding Ca^2+^—signaling proteins, including those able to increase Ca^2+^ in the cytosol, such as the sarcoplasmic Ca^2+^ channel, the ryanodine receptor 2 (Ryr2), the inositol triphosphate receptor (Itpr2, Itpr3, Itpka); and L-type calcium channel (Cacna1d and Ppapdc2), and also the broad spectrum protein regulators, like Ca^2+^/calmodulin-dependent protein kinase II (Calm2, Camk2, Camk4) which, together, can cause smooth muscle cell relaxation [219,220,221,222,223].

A transcriptome analysis of ischemic stress responses following NO_3_^−^ intake indicates the up-regulation of genes enrolled in the lipid and carbohydrate metabolisms and the intracellular transport of molecules, as well as genes related to protein synthesis, turnover, and repair, including those encoding glucokinase, pyruvate dehydrogenase kinase, acetyl coenzyme A acetyltransferase 2, acyl CoA synthetase short-chain,17-dehydrocholesterol reductase, retinol dehydrogenase 11, farnesyl diphosphate synthase, nucleoside transporter, sodium/bile acid co-transporter family member, carbonic anhydrase 3, G2 cyclin, Rho GTPase, activating protein 9, glutamyl aminopeptidase and beta-lactamase 2 [224].

Betanin promotes healthy benefits to the cardiovascular system due to its anti-radical scavenger effect, reducing the reactivity of these molecules, protecting from endothelial tissue from damage. Simultaneously, betanin down-regulates the mRNA of pro-inflammatory mediators while reinforcing endogenous antioxidant defenses. Furthermore, several lines of evidence implicate betanin in the transcriptional regulation of metabolic and antioxidant/detoxification genes [184]. In human hepatic cells, betanin induced translocation of Nrf2 from the cytosol to the nuclear compartment, where it can bind to the antioxidant response element, and, in turn, control mRNA expression and protein levels of several detoxifying/antioxidant enzymes, including glutathione S-transferases, quinone dehydrogenase 1 NAD(P)H dependent and heme oxygenase-1 [186,188,225].

Betanin may, therefore, be a supportive therapeutic alternative to attenuate the main mechanisms involved in CVD without any harmful effects. Although the exact mechanisms by which betanin exerts its cardioprotective role have not been yet fully elucidated, its ability to act directly on ROS/RNS species alongside the induction of the antioxidant and cytoprotective Nrf2-ARE pathway and suppression of the inflammatory NFk-B pathway in CVD can account for all betanin health-promoting benefits [184,226]. Furthermore, betanin is bioaccessible, bioavailable, approved for use in foods in quantium satis, and has not shown any harmful or deleterious effects in animals. Thus, clinical trials should be conducted to determine the effective dose and supplementation regimen to achieve the desired health outcomes in human beings.

## 7. Conclusions

Interventions with dietary NO_3_^−^ from beetroot are reported as affecting cardiovascular and metabolic functions by regulating the gene expression patterns or modulating the activity of proteins and enzymes involved in these cellular processes. The cytoprotective effects of NO-derived from NO_3_^−^-NO_2_^−^/NO pathway may be collectively reinforced by certain bioactive compounds naturally found in beetroot.

PCs and OAs identified at high concentrations in beetroot should also be considered antioxidant defense adjuvants in health promotion and chronic disease prevention. However, the most remarkable compound found in beetroot seems to be betanin. Thus, betanin could be a putative candidate to attenuate the oxidative stress status in humans.

If previously described betanin effects in rodent models are confirmed in humans, it can be expected that short-term betanin intake will be able to attenuate the redox state of human cells by cytoprotective effects, regulating glucose and lipid metabolisms, controlling insulin resistance and lipid peroxidation, and, thus, protecting the cardiovascular system, liver, and kidneys from damage.

## Figures and Tables

**Table 1:** Proximate composition, sugars, NO_3_^−^, NO_2_^−^, and saponin contents of beetroot formulations in 100 g of each product.

Compound	Beetroot Formulations			
	Cereal Bar	Gel	Chips	Juice
Ashes (%)	1.30 ± 0.06 ^b^	2.01 ± 0.13 ^a^	1.00 ± 0.05 ^c^	0.80 ± 0.06 ^d^
Moisture (%)	12.90 ± 0.50 ^b^	76.14 ± 0.48 ^a^	4.66 ± 0.57 ^c^	85.50 ± 0.50 ^a^
Energy (kilocalorie)	325.58 ± 2.5 ^b^	148.50 ± 0.01 ^c^	365.05 ± 2.10 ^a^	94.90 ± 1.70 ^d^
Carbohydrate (g)	62.97 ± 0.97 ^b^	42.62 ± 0.31 ^c^	89.96 ± 0.52 ^a^	22.67 ± 0.40 ^d^
Protein (g)	16.20 ± 0.39 ^a^	3.02 ± 0.09 ^b^	0.97 ± 0.01 ^c^	0.70 ± 0.07 ^c^
Lipids (g)	0.97 ± 1.00 ^a^	0.66 ± 0.01 ^b^	0.14 ± 0.01 ^c^	0.16 ± 0.01 ^c^
Total dietary fibers (g)	4.07 ± 0.14 ^a^	3.71 ± 0.10 ^c^	3.22 ± 0.63 ^b^	0.91 ± 0.31 ^d^
Total sugars (g)	37.72 ± 0.70 ^a^	14.90 ± 0.23 ^c^	18.79 ± 0.13 ^b^	12.11 ± 0.35 ^d^
Fructose (g)	2.79 ± 0.15 ^a^	1.21 ± 0.15 ^b^	1.47 ± 0.11 ^b^	0.86 ± 0.19 ^c^
Glucose (g)	4.71 ± 0.16 ^a^	2.61 ± 0.12 ^b^	2.70 ± 0.11 ^b^	2.45 ± 0.21 ^b^
Sucrose (g)	26.59 ± 0.80 ^a^	11.60 ± 0.13 ^c^	14.62 ± 0.17 ^b^	8.80 ± 0.65 ^d^
Maltose (g)	3.63 ± 0.19 ^a^	0.00 ^b^	0.00 ^b^	0.00 ^b^
NO_3_^−^ (mmol)	14.00 ± 0.05 ^a^	6.30 ± 0.01 ^b^	6.90 ± 0.02 ^b^	4.10 ± 0.01 ^c^
NO_2_^−^ (mmol)	0.20 ± 0.01 ^a^	0.11 ± 0.02 ^b^	0.13 ± 0.02 ^b^	0.10 ± 0.02 ^b^
Betanin (mg·g^−1^)	173 ± 0.12 ^c^	246 ± 0.07 ^b^	1274 ± 0.01 ^d^	298.5 ± 0.03 ^a^

Values are expressed as means ± SD. Different letters within the same line indicate differences between samples at a significance level of *p* < 0.05. Beetroot-cereal bar and gel values are reproduced from Baião et al. [1] and da Silva et al. [12], respectively.

**Table 2:** Organic acids (OA) and phenolic compounds (PC) contents in beetroot product interventions.

Compounds	Beetroot Product Interventions			
	Cereal Bar	Chips	Gel	Juice
OAs (mg/g)				
Citric acid	2.31 ± 0.14 ^a^	1.52 ± 0.06 ^b^	1.04 ± 0.10 ^c^	0.89 ± 0.10 ^d^
Ascorbic acid	1.55 ± 0.21 ^a^	0.93 ± 0.09 ^b^	0.53 ± 0.04 ^c^	0.41 ± 0.03 ^d^
Malic acid	3.00 ± 0.10 ^a^	2.26 ± 0.10 ^b^	1.59 ± 0.01 ^c^	1.34 ± 0.20 ^d^
Fumaric acid	0.81 ± 0.10 ^a^	0.63 ± 0.10 ^a,b^	0.41 ± 0.20 ^b^	0.18 ± 0.10 ^c^
Succinic acid	0.51 ± 0.01	-	-	-
Oxalic acid	0.50 ± 0.15	-	-	-
Total	9.19 ± 0.71 ^a^	5.34 ± 0.35 ^b^	3.57 ± 0.35 ^c^	2.84 ± 0.70 ^d^
PCs (mg/100 g)				
Vanillic acid	13.14 ± 0.11	-	-	-
*p*-Coumaric acid	39.68 ± 1.21	-	-	-
Rosmarinic acid	4.25 ± 0.04	-	-	-
3,4-Dihydroxybenzoic acid	9.97 ± 0.12 ^a^	7.85 ± 0.10 ^b^	5.43 ± 0.81 ^c^	3.79 ± 0.03 ^d^
Gallic acid	60.50 ± 1.76 ^a^	22.49 ± 1.18 ^b^	8.81 ± 0.15 ^c^	4.10 ± 0.06 ^d^
Syringic acid	4.48 ± 0.00 ^a^	4.47 ± 0.01 ^a^	3.78 ± 0.02 ^b^	3.27 ± 0.05 ^b^
Caffeic acid	5.94 ± 0.03 ^a^	3.57 ± 0.06 ^b^	3.34 ± 0.21 ^b^	2.90 ± 0.00 ^c^
Ferulic acid	3.23 ± 0.01 ^a^	0.88 ± 0.04 ^b^	0.82 ± 0.11 ^b^	0.77 ± 0.01 ^b^
Chlorogenic acid	5.69 ± 0.01 ^a^	3.36 ± 0.02 ^b^	3.27 ± 0.12 ^b^	3.17 ± 0.45 ^b^
Total	147.73 ± 3.30 ^a^	42.62 ± 1.39 ^b^	25.45 ± 1.42 ^c^	18.00 ± 0.61 ^d^
Saponins (mg/100 g)	8648.00 ± 1.85 ^a^	6371.00 ± 1.26 ^b^	2200.00 ± 0.17 ^d^	2599.00 ± 1.27 ^c^

Values are expressed as means ± SD. Different letters within the same line indicate differences between samples at a significance level of *p* < 0.05. Beetroot juice, density = 1 mg/mL. OA, organic acids; PC, phenolic compounds. Beetroot-cereal bar values are reproduced from Baião et al. [1].

**Table 3:** Health effects of beetroot supplementation: reports from human intervention trials performed in the last 5 years (2014–2019): Features of beetroot product interventions, supplementation regimen, biochemical and hemodynamic parameters of healthy, physically active or cardiovascular-compromised patients.

Study	Beetroot Product Intervention	Bioactive Compounds	Experimental Population	Trial Features	Biochemical and Metabolic Effects	Hemodynamic Effects
Hobbs et al. [26]	Beetroot bread	NO_3_^−^ (1.1 mmol) NO_2_^−^ (<0.03) Betacyanins (12.1 mg)	Fourteen men genotyped for the Glu298Asp polymorphism in the eNOS gene	Randomized Single-blind Crossover Placebo-controlled Acute ingestion (10 days washout)	↑ plasmatic NO_3_^−^ and NO_2_^−^	↓ clinic DBP iAUC _0–6 h_ (–36 ± 12 mm Hg) ↓ clinic SBP iAUC _0–6 h_ (−29 ± 17 mm Hg)
Jajja et al. [202]	Beetroot juice (70 mL) Blackcurrant juice (70 mL)	NO_3_^−^(≈6.45 mmol) NO_2_^−^ (ND) NO_3_^−^ (≈0.04 mmol) NO_2_^−^ (ND)	Twenty-four older and overweight subjects	Randomized Double-blind Placebo-controlled Chronic ingestion (3 weeks and 1 week washout)	↑urinary NO_3_^−^ and NO_2_^−^ concentration ↑salivary NO_3_^−^ and NO_2_^−^ concentration No changes in NO synthesis after blackcurrant juice ingestion	No changes in resting clinic BP or 24-h ABPM ↓ daily SBP (−7.3 ± 5.9 mm Hg) ↓ BP was not maintained after the interruption of beetroot juice supplementation
Baião et al. [11]	Beetroot juice (100 mL) Nitrate-depleted beetroot juice (100 mL)	NO_3_^−^ (1.60 mmol) NO_2_^−^ (< 0.01 mmol) PCs (17.99 mg) OAs (284 mg) NO_3_^−^ (0.005 mmol) NO_2_^−^ (< 0.01 mmol) PCs (14.22 mg) OAs (241 mg)	Forty healthy subjects with no cardiovascular, pulmonary, and/or metabolic Diseases	Randomized Double-blind Crossover Placebo-controlled Acute ingestion (1 week washout)	↑ urinary NO_3_^−^ and NO_2_^−^ concentrations No changes in NO synthesis between men and women No changes in NO synthesis after placebo juice ingestion	-
Bondonno et al. [203]	Beetroot juice (140 mL) Nitrate-depleted beetroot juice (140 mL)	NO_3_^−^ (7 mmol) NO_2_^−^ (<0.001 mmol) NO_3_^−^ (0.001 mmol) NO_2_^−^ (< 50 nmol)	Twenty-seven treated hypertensive individuals	Randomized Double-blind Crossover Placebo-controlled (1 week ingestion and 1 week washout)	↑plasmatic NO_3_^−^ and NO_2_^−^ concentrations ↑salivary NO_3_^−^ and NO_2_^−^ concentrations ↑ urinary NO_3_^−^ and NO_2_^−^ concentrations	No differences in home BP and 24-h ambulatory BP
Kapil et al. [29]	Beetroot juice (250 mL) Placebo beetroot juice (250 mL)	NO_3_^−^ (≈ 6.4 mmol) NO_2_^−^ (< 50 nmol/L) NO_3_^−^ (≈ 0.007 mmol) NO_2_^−^ (< 50 nmol/L)	Thirty-four drug-naive and 34 treated patients with hypertension	Randomized Double-blind Placebo-controlled Chronic ingestion (4 week and 1 week washout)	↑plasmatic NO_3_^−^ and NO_2_^−^ concentrations ↑plasmatic cGMP concentrations	↓ clinic BP, 24-h ambulatory BP and home BP. No evidence of tachyphylaxis over the 4-weeks intervention period. ↑ endothelial function by ≈20% ↓ arterial stiffness by 0.59 m/s
Velmurugan et al. [204]	Beetroot juice (250 mL) Placebo beetroot juice (250 mL)	NO_3_^−^ (6.0 mmol) NO_2_^−^ (< 50.0 nmol) NO_3_^−^ (0.001 mmol) NO_2_^−^ (< 50.0 nmol)	Sixty-five hypercholesterolemic subjects (32 received placebo and 33 received dietary NO_3_^−^)	Randomized Double-blind Placebo-controlled Chronic ingestion (6 weeks and 1 week washout)	↑ urinary, salivary and plasmatic NO_3_^−^ and NO_2_^−^ concentration No changes in electrolytes (Na^+^, K^+^ and Cl^−^) ↓ platelet-monocyte aggregates ↓ stimulated P-selectin expression	↑ FMD ↓ AIx ↓ aPWV ↓ SBP but not DBP and heart rate
da Silva et al. [12]	Beetroot Gel (100 g)	NO_3_^-^ (6.30 mmol) NO_2_^−^ (0.003 mmol) PCs (24.20 mg) OAs (357 mg) Saponins (3200 mg) Betanin (87 mg)	Five healthy volunteers with no cardiovascular, pulmonary, and/or metabolic diseases	Double-blind Acute ingestion (3 h of data collection)	↑ plasmatic NO_2_^−^ concentration	Minimal effects on brachial SBP and DBP
Vasconcellos et al. [19]	Beetroot gel (100 g) Placebo beetroot gel (100 g)	NO_3_^−^ (9.92 mmol) NO_2_^−^ (< 10 μmol) PCs (27.13 mg) OAs (366 mg) Saponins (3659 mg) Betanin (82 mg) NO_3_^−^ (0.33 mmol) NO_2_^−^ (< 10 μmol) PCs (21.13 mg) OAs (313 mg) Saponins (3059 mg) Betanin (63 mg)	Twenty-five physically active, runners, with no cardiovascular, pulmonary, and/or metabolic diseases	Randomized Double-blind Crossover Placebo-controlled Acute ingestion (1-week washout)	↑ urinary NO_3_^−^ and NO_2_^−^ after 60, BE(T90) and AE concentration ↑ Blood glucose concentrations after AE and +20 (93.95±19.32 mg⋅dL^−1^) No changes in blood lactate, serum cortisol, and urinary urea concentration	No changes in VO_2peak_, time to fatigue, respiratory quotient, SBP, and DBP
Bock et al. [205]	Beetroot powder (10 g) Placebo beetroot powder (10 g)	NO_3_^−^ (≈ 4.03 mmol) NO_2_^−^ (≈ 0.29 mmol)	Thirteen healthy older adults with no cardiovascular, respiratory, or metabolic diseases, non-obese, and non-smokers	Randomized Double-blind Crossover Placebo-controlled 4 weeks ingestion and 1 week washout)	-	↓ ventilatory responsiveness to hypoxia ↓ SBP and mean BP No changes in heart rate responsiveness No changes in spontaneous cardiovagal BRS
Cuenca et al. [206]	Beetroot juice (70 mL) Nitrate-free juice (70 mL)	NO_3_^−^ (6.4 mmol) NO_2_^−^ (≈ 0.04 mmol)	Fifteen healthy resistance-trained men	Randomized Double-blind Crossover Placebo-controlled Acute ingestion (1-week washout)	No changes in blood lactate concentration	↑ peak and mean power output and ↓ time taken to reach W_peak_ in the Wingate test No changes in fatigue index, over time and power CMJ height
de Castro et al. [207]	Beetroot juice (420 mL) Placebo NO_3_^−^-depleted (420 mL)	NO_3_^−^ (8.4 mmol) NO_2_^−^ (ND) NO_3_^−^ (0.01 mmol) NO_2_^−^ (ND)	Fourteen male recreational runners	Randomized Double-blind Crossover Placebo-controlled Acute ingestion (1-week washout)	No changes in La_peak_, La_post_, Gly_pre_, and Gly_post_.	No changes in 10-km running time performance and total MV
Baião et al. [208]	Beet-cereal bar (60 g) Placebo beet-cereal bar (60 g)	NO_3_^−^ (9.57 mmol) NO_2_^−^ (0.12 mmol) PCs (88.60 mg) OAs (551.4 mg) Saponins (8648 mg) Betanin (66 mg) NO_3_^−^ (0.02 mmol) NO_2_^−^ (0.07 mmol) PCs (83.36 mg) OAs (431.2 mg) Saponins (7566 mg) Betanin (48 mg)	Five patients displaying three risk factors for cardiovascular diseases development	Randomized Double-blind Crossover Placebo-controlled 3 weeks ingestion trial and 1-week washout	↑ plasmatic NO_3_^−^ and NO_2_^−^ concentration.	↓ arterial stiffness through AIx, _ao_PP, and PWV ↓ arterial blood pressures ↓ endothelial dysfunction by improvements in cutaneous microvascular conductance peak No changes in endothelial dysfunction, arterial stiffness, and arterial blood pressure after placebo cereal bar ingestion
Bezerra et al. [209]	Beetroot juice (200 mL) Fruit soda (200 mL) Water (200 mL)	NO_3_^−^ (≈ 13.0 mmol) NO_2_^−^ (ND) NO_3_^−^ (≈ 0.08 mmol) NO_2_^−^ (ND) NO_3_^−^ (ND) NO_2_^−^ (ND)	Fourteen non-hypertensive obese males	Randomized Double-blind Crossover Placebo-controlled Acute ingestion (1-week washout)	↑ NOx plasmatic concentration sustained for 1 h post-intervention	No changes in ambulatory SBP and DBP.
Berends et al. [210]	Beetroot juice (70 mL) Beetroot juice + vitamin C (70 mL)	NO_3_^−^ (6.45 mmol) NO_2_^−^ (ND) NO_3_^−^ (6.45 mmol) NO_2_^−^ (ND) Vitamin C (1000 mg)	Twenty-nine recreational sports subjects	Randomized Double-blinded 1 week ingestion and 1 week washout)	↑ urinary NO_3_^−^ and NO_2_^−^ after beetroot juice ↑ urinary NO_3_^−^ and NO_2_^−^ after beetroot juice + vitamin C No changes in urinary vitamin C excretion in both interventions Beetroot juice + vitamin C ingestion inhibited *N*-nitroso compounds increases	-
Husmann et al. [211]	Beetroot juice (70 mL) Placebo NO_3_^−^depleted	NO_3_^−^ (6.5 mmol) NO_2_^−^ (ND) NO_3_^−^ (0.04 mmol) NO_2_^−^ (ND)	Twelve recreational active males	Randomized Double-blind Crossover Placebo-controlled Ingestion for 5 days (1-week washout)	-	↑ time-to-exhaustion ↓ both lower ΔMVT and ΔPS100 ↓ perception of effort and leg muscle pain
Kim et al. [212]	Beetroot juice (140 mL) NO_3_^−^-depleted beetroot juice (140 mL)	NO_3_^−^ (9.7 mmol) NO_2_^−^ (< 50.0 nmol) -	Thirteen healthy post-menopausal and 10 pre-menopausal women	Randomized Double-blind Placebo-controlled Acute ingestion (1-week washout)	↑ plasmatic NO_3_^−^ and NO_2_^−^ concentrations after 100 min and at the end of the study	Brachial and derived-aortic variables showed the expected age-associated differences in these women ↓ brachial SBP, brachial mean BP, aSBP, and mean aBP and ↑ PP amplification
Kukadia et al. [213]	Beetroot juice (70 mL) Nitrate-free juice (70 mL)	NO_3_^−^ (7.3 mmol) NO_3_^−^ (< 0.06 mmol)	Fifteen healthy subjects with no hypertension or any medication (other than an oral contraceptive pill)	Randomized Double-blind Crossover Placebo-controlled Acute ingestion (1-week washout)	-	Minimal effects on brachial BP and cfPWV. No sustained changes in aortic SBP over subsequent 24 h No sustained changes in hemodynamic parameters during ambulatory monitoring.
Ritz et al. [214]	Beetroot juice (70 mL) No-beetroot control (70 mL)	NO_3_^−^ (6.5 mmol) -	Sixty healthy subjects (16 volunteers with asthma)	Single-blind Randomized Placebo-controlled 1-week ingestion and 1-week washout	-	↓ cold symptom severity and global sickness during and after final exams Healthy vs asthma group interaction was significant for cold symptom severity and global sickness, indicating that the advantage of the beetroot juice group was greater for participants with asthma than for healthy volunteers.
Rokkedal-Lausch et al. [215]	Beetroot juice (140 mL) NO_3_^-^-depleted beetroot juice (140 mL)	NO_3_^−^ (12.4 mmol) NO_3_^−^ (≈ 0.001 mmol)	Twenty healthy male cyclists	Randomized Double-blinded Counter balanced-crossover Placebo-controlled 1-week ingestion and 1 week washout	↑ plasmatic NO_3_^−^ and NO_2_^−^ concentrations prior to time trial tests in normoxia and hypoxia conditions. ↑ TT performance with no difference between normoxia and hypoxia.	↑ VO_2_ and VE during TT, with no difference between normoxia and hypoxia. No changes in heart rate, oxygen saturation, or muscle oxygenation during TT.
Jones et al. [216]	Beetroot juice (70 mL) Prune juice (70 mL)	NO_3_^−^ (6.45 mmol) NO_2_^−^ (ND) NO_3_^−^ (< 0.01 mmol) NO_2_^−^ (ND)	Twenty older subjects	Randomized Double-blinded Placebo-controlled Chronic intervention (2 weeks ingestion and 1-week washout	↑ plasmatic NO_3_^−^ concentrations No changes in endothelium-dependent (by Ach administration) or endothelium-independent (by SNP administration) microvascular responses between groups	↓ SBP by −6±7 mm Hg and DBP by −4±3 mm Hg ↓ SBP by −4±10 mm Hg and DBP by −2±6 mm Hg after the 4 weeks treatment ↑ FMD values by 1.5%±1.8% after 4 weeks
Smith et al. [217]	Beetroot juice (70 mL) Nitrate-depleted placebo (70 mL)	NO_3_^−^ (≈6.2 mmol) NO_2_^−^ (ND) NO_3_^−^ (< 0.004 mmol) NO_2_^−^ (ND)	Twelve recreational trained male university students	Randomized Double-blind Crossover Placebo-controlled Acute ingestion (1-week washout)	-	No changes on sprint performance and total work done in either temperate or hot, humid conditions. No changes between trials for tympanic temperature measured at the conclusion of the exercise trial ↓ peak and mean power output in the hot and humid conditions

ABPM, ambulatory blood pressure mean; Ach, acetylcholine; AE, after exercise; AIx, augmentation index; _a_BP, aortic blood pressure; _ao_PP, aortic pulse pressure; _a_PWV, aortic pulse wave velocity; _a_SBP, aortic systolic blood pressure; BE(T90), before exercise (time 90 min); BP, blood pressure; BRS, baroreflex sensitivity; _cf_PWV, carotid-femoral pulse wave velocity; GMP, cyclic guanosine monophosphate; Cl^−^, chloride; CMJ, countermovement jumps; DBP, diastolic blood pressure; FMD, mediated flow dilatation; Gly_pre_, pre-test glucose concentration; Gly_post_, post-test glucose concentration; K^+^, potassium; La_peak_, peak lactate concentration; mm Hg, millimeter of mercury; MV, mean velocity; MVT, maximal voluntary torque; Na^+^, sodium; ND, not detected; NO, nitric oxide; NOx, nitrate + nitrite concentration; NO_2_^−^, nitrite; NO_3_^−^, nitrate; OAs, organic acids; PCs, phenolic compounds, PP, pulse pressure; PS, paired electrical stimuli; SBP, systolic blood pressure; SNP, sodium nitroprusside; TT, time trial; VE, ventilation expired; VO_2peak_, peak maxim oxygen volume, ND—not determined.
